# Dynamics of respiratory virus spread indoors suggest a holistic approach for exposure risk mitigation

**DOI:** 10.1017/ash.2025.10147

**Published:** 2025-10-29

**Authors:** M. Khalid Ijaz, Syed A. Sattar, Stephanie A. Boone, Raymond W. Nims, Julie McKinney, Charles P. Gerba

**Affiliations:** 1 Global Research & Development for Lysol and Dettol, Reckitt Benckiser LLC, Montvale, NJ, USA; 2 Department of Biochemistry, Microbiology & Immunology, Faculty of Medicine, University of Ottawa, Ottawa, ON, Canada; 3 Department of Environmental Science, University of Arizona, Tucson, AZ, USA; 4 Syner-G Biopharma, Boulder, CO, USA

## Abstract

The severe acute respiratory syndrome coronavirus 2 (SARS-CoV-2) pandemic represented a time of mixed messaging around the relevance of various respiratory virus transmission pathways, with debate extending from the regional health authorities to individual subject matter experts around the world. Globally, the non-pharmaceutical interventions (NPIs) for dealing with the pandemic virus were, fortunately, based on the potential risk posed by each of the transmission pathways (direct droplet transmission, transmission by aerosolized virus, and indirect transmission via surfaces/hands or re-aerosolization of infectious virus from contaminated environmental surfaces). These NPIs were very effective in limiting transmission of SARS-CoV-2 and other respiratory viruses. We believe that there are numerous interdependencies between the various virus transmission pathways, indicating the need for a more holistic consideration of the importance of all transmission pathways and the corresponding need for targeted NPIs during virus outbreaks and pandemics.

On the basis of knowledge gained with other respiratory viruses, such as SARS-CoV and influenza viruses, direct exposure to contaminated infectious respiratory droplets has been considered the primary transmission mechanism for SARS-CoV-2 and most respiratory viruses.^1^ Statements from various regional health authorities have reiterated this opinion, virtually deprioritizing the significance of aerosol transmission and indirect transmission via contaminated fomites for spread of respiratory viruses.^2^

Fortunately, when regional health authorities across the globe stipulated the directives/guidelines of NPIs to be followed during various stages of the pandemic, those guidelines were based on a holistic outlook on the relevance of the different modes of virus transmission.^3^ For instance, mask or respirator wearing was intended to mitigate risk from airborne viruses in general (ie, viruses contained both in droplets and aerosols), and the emphasis on hand washing was intended to mitigate transfer of infectious virus from contaminated surfaces to a susceptible person’s mucous membranes. Testing and isolation of virus-positive individuals, stay-at-home orders, business and school closures, and avoidance of crowded venues in general each recognized the fact that putting people in crowded situations increases risk of exposure and virus spread.

The effectiveness of those NPI guidelines for reducing infection spread translated not only into limiting the spread of SARS-CoV-2 but, importantly, led to decreased spread of other seasonal respiratory infections associated with enveloped viruses (eg, influenza viruses, respiratory syncytial virus, parainfluenza viruses, human metapneumovirus, and other human coronaviruses such as HCoV-229E and HCoV-OC43).^3^ The NPI directives also were found to limit the spread of non-enveloped respiratory viruses (eg, adenoviruses and rhinoviruses).^3^

## Interdependencies between respiratory viral transmission pathways

During the pandemic years, why were the globally implemented NPIs so successful in limiting the spread of a variety of respiratory viral infections, and not just SARS-CoV-2? We believe this is due to the fact that these NPIs, as mentioned above, were intended to address each of the viral transmission pathways, not just the droplet pathway.^3^ Instead of being mutually exclusive, the three virus transmission pathways (involving infectious droplets, aerosols, virus-contaminated fomites and hands) display interdependencies.^2,4–6^ For instance, respiratory droplets produced during a human expiratory event (sneezing, coughing, talking, or breathing) can directly infect another individual who is at close range or can evaporate to form smaller droplets or aerosols.^1,2,4–6^ Alternatively, these droplets may experience hygroscopic growth to increase in mass.^1,4^ Gravitational settling of virus-contaminated droplets may result in contamination of environmental surfaces (fomites),^7,8^ which may then exchange infectious virus to the hand of an uninfected individual^7,8^ leading to transfer to a susceptible mucous membrane and subsequent initiation of an infection.

Viruses deposited on environmental surfaces through settling of droplets or by exchange from contaminated hands^9,10^ may be re-aerosolized (re-suspended) through human activities such as walking, vacuuming, or flushing toilets, to contribute to airborne virus load including association with aerial particulate matter such as dust and allergens.^2,11–14^ The stability of airborne respiratory viruses depends on a number of factors, including the ambient temperature and relative humidity (RH), but also the matrix (pathophysiological, such as mucous, or non-physiological, such as PM_2.5_, pollen or dust) that the virus associates with.^2,15–19^

**Figure 1.**
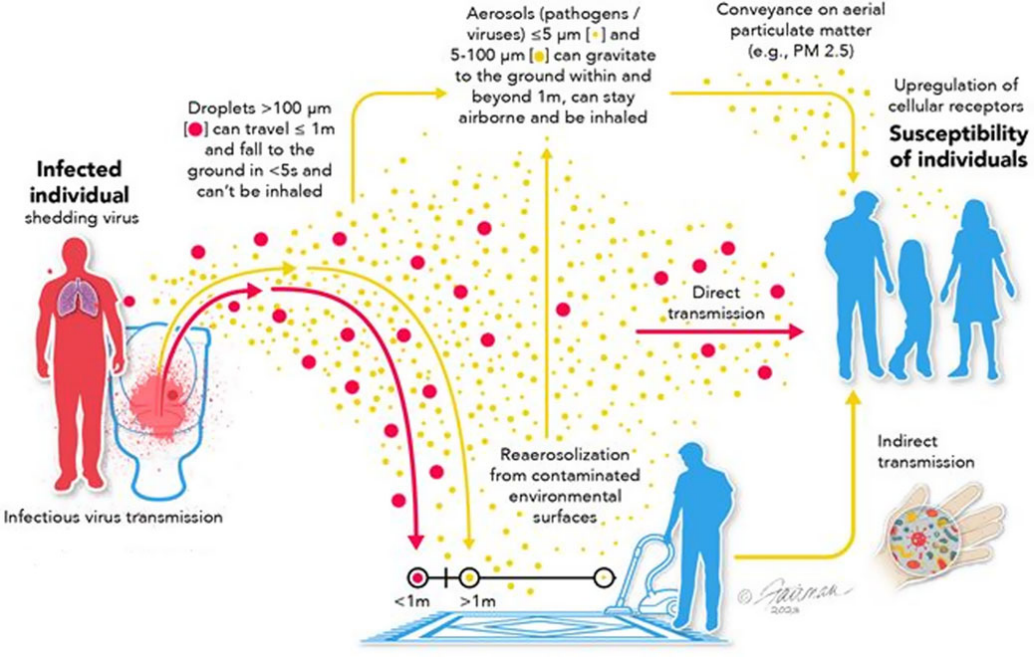
Schematic depiction of major virus transmission pathways, indicating interdependencies. Image source:^2^ CC BY 4.0. https://creativecommons.org/licenses/by/4.0/.

These interdependencies, which may not be generally acknowledged, are depicted schematically in Figure 1. It is understood by the authors that more robust empirical supporting evidence for the biological significance of these proposed interdependencies must be collected. In particular, more clarity on the amounts of infectious virus (as opposed to RNA) released by an infected individual during breathing, talking, sneezing, or coughing, or re-aerosolized by mechanical activities such as vacuuming, walking, or removing clothing, must be obtained.^2^ The challenges associated with air sampling for infectious virus have been reviewed^2^ and must be overcome in order to answer many of the remaining questions on the relevance of certain of the interdependencies discussed above (especially the importance of virus re-aerosolization from fomites and the association of infectious virus with aerial particulates). Evidence of virus spread via the air/surface/hand nexus has been summarized as a workshop consensus outcome by Abney et al.^20^

## A more holistic outlook on respiratory viral transmission

The fact that the NPIs implemented during the SARS-CoV-2 pandemic were so effective at limiting respiratory virus spread in general, and not just SARS-CoV-2,^3^ is consistent with, but not necessarily proof of, a hypothesis that the transmission of respiratory viruses depends on pathways in addition to respiratory droplet transmission. Viral infection transmission may best be viewed as resulting from a dynamic virus spread from infected individuals to susceptible individuals by a spectrum of physical states of active respiratory emissions, instead of the current paradigm that emphasizes separate dissemination by respiratory droplets, aerosols, or by contaminated fomites. For instance, the holistic outlook recognizes the exposure risk associated with the short-distance, short-time exposure to larger respiratory droplets that theoretically^1,15,21^ contain a higher concentration of viral particles that have been exposed to less time for inactivation within the droplet matrix. Alternatively, the holistic outlook acknowledges that viruses may persist (remain infectious) for some time in aerosols.^2,17,18^ For instance, the infectivity half-life of SARS-CoV-2 aerosolized in physiological (artificial saliva), or non-physiological (tissue culture medium) matrices has been reported to be 42 and 75 minutes, respectively, at 40–60% RH.^17^ The extensive literature on persistence of SARS-CoV-2 on surfaces has been reviewed by Ijaz *et al*.^22^ As an example, the half-life of the virus deposited on plastic or stainless-steel surfaces in the presence of a physiological matrix (human sputum or mucus or an organic soil load) has been reported to be 3.1 or 29 hours, respectively.^23,24^ On LabSkin, the half-life of SARS-CoV-2 has been reported to be 2.8 to 17.8 hours, depending upon the temperature.^25^

This persistence of infectious virus in air and on surfaces suggests that long-distance, extended-time transmission through infectious respiratory viral particles associated with aerosols (either derived from droplets or from re-aerosolization from contaminated fomites) also might lead to infection of susceptible persons sharing indoor spaces with an infected individual. Finally, the exposure risk associated with indirect transmission from fomites to new hosts involves more than the simple exchange among contaminated surfaces to hands to susceptible host mucous membranes (ie, the surface/hand/mucous membrane nexus). The recognition^2,10–12^ that re-aerosolization of viruses from fomites to aerosols occurs may necessitate reassessment of the risk associated with the indirect (ie, fomite) transmission pathway.

## Approaches for mitigation of risk should consider each of the relevant transmission pathways

As mentioned above, different NPIs may apply to different transmission pathways (reviewed in reference 2). For instance, the wearing of a face mask or respirator is intended to mitigate risk of transmission of respiratory viruses through the droplet route, especially when worn by presymptomatic, asymptomatic, or symptomatic individuals. To a certain degree, these masks or respirators may help to mitigate risk of acquiring an infection through inhalation of airborne viruses, in general (ie, viruses in droplets or aerosols). However, these devices also reduce the frequency of face touching, thereby reducing the risk of exposure of oral and nasal mucosa to virus transmitted via the hands by touching contaminated fomites. Air decontamination within indoor spaces may mitigate risk associated with airborne respiratory viruses, either associated with droplets or with aerosols and whether associated with respiratory excretions/secretions originating from an infected person or with environmental dust, pollen, or other pollutants such as PM_2.5_. This may be accomplished through use of air-sanitization technologies, or by increasing the room ventilation by opening of windows and/or increasing the rate of air exchange^26^ through heating and air ventilation system upgrades. Crowded indoor spaces favor, for a variety of reasons, the accumulation of viruses in air and on environmental surfaces, hence the advisability of restrictions in school, business, and hospitality venues during outbreaks and pandemics. Risk of indirect exposure to viruses through the hand/surface/mucous membrane nexus is best mitigated through increasing the frequency of hand washing or use of appropriate hand-sanitizing agents. In addition, the accumulation of viruses on environmental surfaces may be reduced through the targeted use of surface hygiene agents, implementation of surfaces with continuous disinfecting characteristics,^27^ or the use of air sanitization technologies.^28^ Remaining mindful of the potential for re-aerosolization of viruses through human activities is necessary for mitigating exposure risk associated with each of the transmission pathways. Even the removal of personal protective equipment such as face masks or respirators, gloves, or gowns may lead to virus re-aerosolization.^29,30^

## Significance

The World Health Organization has highlighted the point that it is difficult to separate potential direct and indirect exposure modes in establishing virus transmission relevancy,^31^ and this difficulty contributes greatly to the debate mentioned above regarding the relative importance of the various transmission pathways. Bringing infected and noninfected individuals (including pre or asymptomatic cases) into proximity necessarily increases the opportunities for indoor air contamination with droplets/aerosols, subsequently contaminating fomites within the immediate area. Stated another way, the same factors that increase exposure risk associated with one of the virus transmission pathways necessarily increase the risk associated with the other pathways. Realizing the various interdependencies holistically among the different pathways, it is more challenging to proactively determine the most important factors for virus transmission and, therefore, the optimal bundle of NPIs to use for exposure risk mitigation.

A more holistic outlook on virus transmission is also informed by the concept of a continuum of sizes for droplets and aerosols, and the importance of residence time over which viruses remain airborne and infectious in droplets, aerosols, and particulate matter. The particulates of concern include gravitationally settled particles, which contaminate environmental surfaces, as well as those potentially becoming re-aerosolized from those surfaces ending up as airborne viruses that might be inhaled or land on and secondarily contaminate other environmental surfaces.

It would be beneficial to public health if individual and group biases regarding the perceived importance of the various virus transmission pathways could be set aside and a more holistic outlook encompassing the truly multimodal nature of respiratory virus dissemination could be acknowledged more broadly. In this manner, a science-based public health advisory decision-making process that takes into account the interdependencies discussed here might be implemented. In a practical sense, the global responses to the SARS-CoV-2 pandemic correctly considered most of the modes of transmission discussed herein during implementation of regionally mandated NPIs intended to limit viral dissemination. This should be considered going forward and taken into account in the messaging from health authorities, to limit confusion about sources of transmission risk.

The World Health Organization has recently^31^ done a comprehensive job of acknowledging the various transmission modes for SARS-CoV-2. Their risk assessment methodology takes into account: [1] the opinion that most respiratory viruses spread through zoonotic transmission, *direct* (body surface-to-body surface) *contact* and *indirect contact* (fomite) transmission, *direct deposition* (droplet) transmission, and *inhalation* (short-range) or *airborne* (long range, aerosol) transmission; [2] acknowledgment that persistence of infectious virus in air is dependent on factors such as the matrix with which the virus is associated, temperature, RH, and ultraviolet radiation; [3] the relatively low minimum human infectious dose for SARS-CoV-2 of ∼10 tissue culture infectious doses (TCID_50_); [4] the challenges associated with sampling of indoor air and fomites for recovering infectious SARS-CoV-2; and [5] the relationship between empirically determined in-air concentrations of SARS-CoV-2 RNA versus infectious SARS-CoV-2. We believe that this is a step in the right direction. Until we have definitive data to rule out one or more of the transmission modalities, considering the potential interdependencies among the various modalities, we should not discount the importance of any specific modalities and associated NPIs in our official public health messaging.
